# Vaccine Candidates against Coronavirus Infections. Where Does COVID-19 Stand?

**DOI:** 10.3390/v12080861

**Published:** 2020-08-07

**Authors:** Jawad Al-Kassmy, Jannie Pedersen, Gary Kobinger

**Affiliations:** 1 Department of Experimental Surgery, McGill University, Montreal General Hospital 1650 Cedar Avenue, Montreal, QC H3G 1A4, Canada; 2 Axe des Maladies Infectieuses et Immunitaires, Centre de Recherche du Centre Hospitalier Universitaire de Québec-Université Laval, Québec, QC G1V 4G2, Canada; jannie.pedersen.1@ulaval.ca (J.P.); gary.kobinger@crchudequebec.ulaval.ca (G.K.); 3 Département de Microbiologie-Infectiologie et Immunologie, Faculté de Médecine, Université Laval, Québec, QC G1V 0A6, Canada; 4 Department of Medical Microbiology, University of Manitoba, Winnipeg, MB R3E 0J9, Canada; 5 Department of Pathology and Laboratory Medicine, University of Pennsylvania School of Medicine, Philadelphia, PA 19104-4238, USA

**Keywords:** coronavirus, COVID-19, MERS, SARS, vaccine, clinical trials

## Abstract

Seven years after the Middle East respiratory syndrome (MERS) outbreak, a new severe acute respiratory syndrome coronavirus 2 (SARS-CoV-2) made its first appearance in a food market in Wuhan, China, drawing an entirely new course to our lives. As the virus belongs to the same genus of MERS and SARS, researchers have been trying to draw lessons from previous outbreaks to find a potential cure. Although there were five Phase I human vaccine trials against SARS and MERS, the lack of data in humans provided us with limited benchmarks that could help us design a new vaccine for Coronavirus disease 2019 (COVID-19). In this review, we showcase the similarities in structures of virus components between SARS-CoV, MERS-CoV, and SARS-CoV-2 in areas relevant to vaccine design. Using the ClinicalTrials.gov and World Health Organization (WHO) databases, we shed light on the 16 current approved clinical trials worldwide in search for a COVID-19 vaccine. The different vaccine platforms being tested are Bacillus Calmette–Guérin (BCG) vaccines, DNA and RNA-based vaccines, inactivated vaccines, protein subunits, and viral vectors. By thoroughly analyzing different trials and platforms, we also discuss the advantages and disadvantages of using each type of vaccine and how they can contribute to the design of an adequate vaccine for COVID-19. Studying past efforts invested in conducting vaccine trials for MERS and SARS will provide vital insights regarding the best approach to designing an effective vaccine against COVID-19.

## 1. Introduction

History always holds valuable lessons for us. As the influential Chinese philosopher Confucius once said, “Study the past if you want to define the future”. Now, we certainly know that the novel severe acute respiratory syndrome coronavirus 2 (SARS-CoV-2) is not entirely “novel” itself. Multiple coronaviruses have been discovered in the past years [1]. Today, out of seven types of coronaviruses known to infect humans, only three are considered to be highly pathogenic: the SARS-CoV discovered in 2002 to be the cause of severe acute respiratory syndrome (SARS), the MERS-CoV discovered in 2012 as the responsible virus for the Middle East respiratory syndrome (MERS), and the current SARS-CoV-2 causing Coronavirus disease 2019 (COVID-19). The good news is that we find many similarities between the three coronaviruses. A review by Wang et al. demonstrated the structural and immunological aspects of SARS-CoV-2 similar to SARS-CoV. For example, they found that immunodominant epitopes in SARS-CoV, such as the Spike protein S, are highly conserved in SARS-CoV-2 and have the potential to evoke a T cell response [2]. Scientists and researchers can then leverage these similarities to find therapeutic approaches more easily. While these viruses all belong to the same *Betacoronavirus* genus, use animal reservoirs, and share clinical features upon infection, there still exist essential differences. The questions to be asked today are as follows: What can we learn from the last two coronavirus outbreaks? Can we find similar patterns between SARS, MERS, and COVID-19? If so, how can this help us in developing a new vaccine? The purpose of this review is to outline the development of vaccine candidates against the coronavirus infections since the emergence of SARS, followed by MERS, and recently COVID-19.

## 2. A Historical Overview

In 2002, the emergence SARS in Guangdong, China sparked a significant moment in history. The epidemic affected more than 26 countries globally, with 8000 cases and almost 800 deaths. Symptoms recorded were influenza-like with fever, chills and dry cough, which in 5–7 days progressed to a severe respiratory insufficiency and, in some cases, death [3]. The most vulnerable population was the elderly, with death rates reaching over 50%. It is believed that the coronavirus was transmitted from bats to civets, and finally to humans [1].

Ten years later, a new type of coronavirus, MERS-CoV, emerged in Saudi Arabia, causing the MERS outbreak. MERS-CoV was found to be hosted in bats and camels and was then transmitted to humans [1]. Most cases of MERS required hospitalization owing to severe respiratory disease with typical symptoms of fever, dyspnea, and cough. Like SARS, MERS became a global crisis, affecting 27 countries around the world with a mortality rate of 35% [4]. Both SARS and MERS were put on the World Health Organization (WHO) list of blueprint diseases [5].

In 2019, eight years after MERS, a new type of coronavirus emerged, SARS-CoV-2. The virus made its first appearance in December 2019 in an exotic food market (“Wet Market”) in Wuhan, China. Despite rapidly implemented quarantine measures, the virus spread globally and, in only three months, the number of cases of COVID-19 breached 1 million, with more than 50,000 deaths.

## 3. Similarities between SARS-CoV, MERS-CoV, and SARS-CoV-2 in Areas Relevant for Vaccine Design

The three beta-coronaviruses (CoVs) are all zoonotic agents that emerged from a natural animal reservoir believed to be bats. Through different intermediaries, animal hosts were able to infect humans [1,6]. Phylogenetically, SARS-CoV belongs to linkage B, while MERS-CoV cluster with other bat-derived viruses belong to linkage C [1]. SARS-CoV-2 cluster and other bat-derived CoVs are genetically more closely related to SARS-CoV than MERS-CoV [7].

CoVs have a large genome of 26–32 kb with an overall similarity in the organization of the genome. In the 5′ end of the genome, two open reading frames (ORF) encode for non-structural proteins, while the 3′ end encodes for the Spike protein (S), envelope protein (E), membrane protein (M), and nucleocapsid (N) [1,6,7].

The S protein has been proposed as one of the most promising candidates for vaccine design for the SARS-CoV thanks to its ability to induce neutralizing antibodies and a strong T cell response [8,9]. The S glycoprotein is divided into two subunits, S1 and S2, where S1 contains the receptor-binding domain (RBD), including smaller receptor-binding motif (RBM), while the fusion protein is located in S2. Both SARS-CoV and SARS-CoV-2 bind to the angiotensin-converting enzyme 2 (ACE-2) as the primary target, whereas the predominant cellular receptor for MERS is dipeptidyl peptidase 4 (DPP4) [10]. Lan et al. have demonstrated that the crystal structure of the RBD of SARS-CoV-2 is almost identical to that of SARS-CoV [11]. However, several key residues within the domain vary between the two viruses, and a 10–20-fold higher affinity to the ACE-2 receptor has been observed for SARS-CoV-2, stressing one of the potential impacts of these variations [7,12]. Antibodies often target the RBD, and several SARS-CoV specific monoclonal antibodies (mAbs) have previously been isolated [12,13,14,15]. While many fail to cross-neutralize the SARS-CoV-2, some mAbs targeting the RBD were able to neutralize both viruses, making it a potentially interesting choice for vaccine design with a dual target. Nevertheless, the non-RBD Spike epitopes might be more immunogenic and have greater surface accessibility compared with RBD epitopes with bioinformatic analysis showing that the majority of high-score epitopes are located outside this domain [16]. Ten selected high-score epitopes in SARS-CoV-2 showed epitopes in the N-terminal region (NTD), the C-terminal of S1, the N-terminal of S2, the fusion protein (FP), and heptad repeat domain 2 (HR2). Only two were located in the RBD, and only one was considered conserved (located in the HR2). The HR2 region has previously been proposed as a pan-CoV antiviral inhibitory target, and SARS-CoV studies have shown antibodies targeting this region have efficiently neutralized the virus in vitro, suggesting this domain might be interesting for a vaccine [17,18]. A recent cryogenic electron microscopy (cryo-EM) analysis of the SARS-CoV Spike protein after fusion also emphasizes the potential of the more conserved S2, including the HR2 region, for vaccine developments for a wide range of SARS-like CoV [19]. The S1 region of the SARS-CoV-2 Spike protein, including the NTD, only shares 64% sequence identity with SARS-CoV compared with 91% within the S2 region; however, several predicted high-score epitopes were located in the NTD [14,19]. This region’s potential for vaccine designs has been further established by a research team reporting neutralizing Antibodies (nAb) from recovered COVID-19 patients targeting the NTD [20]. Even though these regions in S1 are less likely to be conserved, the antigenic potential might be superior to other conserved domains in S2. While the S protein is by far the most studied region for vaccine designs, in silico analyses of potential immunogenic epitopes in the SARS-CoV-2 have suggested several domains within the N, M, and E protein as well as the non-structural proteins primarily focusing on T cell responses [21,22,23,24,25]. Using previously generated data from SARS-CoV with experimentally validated epitopes, seven areas were found within SARS-CoV-2 with 100% identity to SARS epitopes and an 85% world population coverage [23]. Of these, two were found in the M protein and one in the N protein. Lee et al., using a similar approach, also identified SARS-CoV-identical epitopes, with the majority being located in the N protein [21]. Kiyotani et al. compared Human Leucocyte Antigens (HLA) I and II SARS-CoV-2 derived epitopes to both MERS-CoV and SARS-CoV and found a substantial number of HLA-I epitopes shared by SARS-CoV and SARS-CoV-2 in both structural and non-structural proteins, whereas only epitopes located in ORF1ab were shared between all three viruses [24]. The lack of shared T cell epitopes within all human CoV structural proteins has previously been illustrated by Liu et al., who found no conserved T cell epitopes between MERS-CoV, SARS-CoV, and two other human coronaviruses HCoV-OC43 and HKU1 [22]. Nevertheless, an informatics approach comparing the more conserved E protein of SARS-CoV-2 with taxonomically related CoV including non-human CoV suggests that some major antigenic epitopes in the envelope might play an important role and could be significant in potential partial protection arising from human–animal interaction [25]. Considering the risk of a future novel CoV appearance, an approach looking at conserved areas within the same cluster of CoV might be preferable for a more broadly protecting vaccine; nonetheless, considering the current pandemic, specific SARS-CoV-2 highly immunogenic targets might be preferable.

## 4. Protective Vaccines against MERS-CoV and SARS-CoV in Animal Models

Since the first outbreak in 2002, substantial work has been done in order to find protective vaccines against emergent coronaviruses, with several early predictions that another novel viral agent would emerge [1,26,27]. Various platforms have been evaluated and, while the majority were able to induce some level of antibodies and T cell responses, sterilizing immunity was not reported in several different animal models [28,29,30,31]. The Spike protein was the most extensively evaluated protein against both SARS-CoV and MERS owing to its immunogenic properties. Using various platforms such as viral vectors, nucleic acids, and subunits, the protein was shown to be able to induce potent humoral and cellular immune responses, which, in many cases, lead to some degree of protection or diminished viral shedding [28,29,30,31]. The advantage of utilizing the whole Spike compared with only S1, RBD, or other subunits has been debated [31,32,33,34,35,36]. Looking at SARS, several vaccines using different viral vectors or DNA were able to induce high levels of neutralizing antibodies using the full-length S protein, which, in some models, provided protection against challenge [36,37,38,39,40]. However, increased liver pathology was also reported in vaccinated animals after challenge, pointing to the risk of antibody disease enhancement (ADE) when utilizing the full-length Spike [41]. This has led to several studies looking at protection following vaccination with various subunits of Spike, including the S1 and RDB with promising results [8,42,43]. Similar considerations have been made in the quest of a MERS-vaccine. The use of viral vectors, nanoparticles, proteins, DNA with the full-length Spike, or subunits like RBD and S1 protections has been observed to various degrees [34,35,44,45,46,47]. In one study, increased lung hemorrhage was observed in animals vaccinated with S1; however, other groups reported no increase in lung pathology in vaccinated groups. Even with the full-length Spike underlining the risk of ADE, using full-length or subunits of the Spike protein would need further evaluation [32,45].

A few groups have examined the effect of non-Spike proteins in challenge models against SARS-CoV [37,48,49,50]. While the protective efficacy was only slightly increased by the addition of M and E to the S vaccine using a parainfluenza virus type 3 vector, no protection was observed without the S protein, underlining the importance of this protein [49]. Immunogenicity of MERS-CoV specific N vaccine has been shown, but the protective role has yet to be evaluated [51]. However, a study using Venezuelan equine encephalitis replicons encoding a SARS-CoV CD4^+^ T cell epitope conserved between SARS-CoV and MERS-CoV showed protection from a lethal challenge dose through interferon (IFN)-g production, implying the possible role for other antigens that are more conserved between the different coronaviruses in a vaccine strategy [50].

When comparing different vaccine platforms, one study looked at the inactivated vaccines and adenovirus vectors expressing the S protein, or the N protein of SARS reported increased protection when utilizing the whole inactivated virus. They argued that, perhaps, the exposure of several proteins would aid the immunogenic response [37,48]. However, historically, the inactivated vaccine produces a weaker immune response in humans, necessitating several prime-boost vaccinations for a sufficient response, making this a potentially less attractive platform [52]. Besides, using a whole inactivated MERS-virus produced a hypersensitive reaction in the lungs, highlighting the possible adverse events with inactivated vaccines [53].

The other vaccine platform tested in the study from See et al. was the adenoviral vector. For the whole inactivated vaccine, the immunogenic response was subsidiary, but a significant reduction in viral titers was reported in mice for both vaccines [48]. Moreover, van Doremalen et al. tested the adenovirus-vectored vaccine ChAdOx1nCoV-19, which encodes the S protein of SARS-CoV-2, in mice and in rhesus macaques. In mice, profiling of IgG subclasses demonstrated a predominantly Th1 response post vaccination and neutralizing antibodies were detected in all mice vaccinated with ChAdOx1nCoV-19. As for rhesus macaques, they found that, compared with control animals, a single vaccination significantly reduced the viral load in bronchoalveolar lavage fluid and respiratory tract tissue [54]. The same group also previously demonstrated that a single dose of ChAdOx1 MERS, a chimpanzee adenovirus vector encoding the S protein of MERS, protected non-human primates against MERS-CoV [55]. In general, there are several advantages to utilizing viral vectors, such as the possibility of long-term gene expression, the high specificity of gene delivery to target cells, and the high immunogenicity after just one vaccination. That being said, the risk of pre-existing immunity against adenoviruses decreasing the desired immunogenic response against the protein of interest is a concern [56].

The DNA platform received increased attention over the years owing to its overall safety profile and high adaptability, followed by a fast-large-scale production [57]. Concerns remain regarding the lower generated immunogenicity and the need for several prime-boost regimes; however, several improved administration techniques and vector optimization have made this a valuable vaccine platform for emergent pathogens [58].

Several studies have looked at the DNA vaccines as a platform for CoV with encouraging results [36,59,60]. Wang and colleagues showed that DNA expressing the full Spike combined with S1 protein was superior to protein alone, reducing lung damage in a Non-Human Primate (NHP) model [59]. Moreover, they showed higher immunogenicity using a DNA–protein regime compared with DNA or protein alone and underlined the advantage of including a different platform for the optimal response.

Finally, live-attenuated SARS-CoV and MERS-CoV viruses have been studied in mice models [61,62]. A group of researchers attenuated the E protein in SARS-CoV and tested the effects in mice. Interestingly, they found that mice vaccinated with the attenuated virus had a significant reduction in the number of proinflammatory cytokines associated with lung injury, as well as increased CD4^+^ and CD8^+^ T cell counts [63]. Genetically modified viruses (lacking the envelope gene or the *NSP16* gene, respectively) were shown to be able to protect mice from infection using both heterologous and homologous viruses. Given the lack of sterilizing immunity from other platforms, some groups suggest this to be a possible way forward [28]. That being said, the risk for clinical disease in immunocompromised people, combined with the risk of the vaccine reconverting back into its pathogenic forms, has made this option less prioritized [52]. Nonetheless, scientists and researchers worldwide must find the right balance between inducing an adequate immune response and assuring the safety of the vaccine.

The optimal antigen with the right platform is crucial; however, another interesting point shown by previous studies is the potential improved protection using mucosal vaccine administration [1,44,50,64,65]. One study comparing intramuscular (IM) with intranasal administration (IN) showed a better local IgA response along with improved systemic T cell response in a mice model [65]. In the study, both administration methods protected from infection. In another study, no virus was found in the lungs of IN-vaccinated mice, while the same vaccine administered IM and controls did not prevent viral amplification in lung tissue [64]. Other groups have looked at lung damage and found that, although protection in both IN and IM was observed, the changes in the lungs were less in the IN-administered group [48]. They also pointed out that, in the IN group, serum nAb titers were lower, indicating that the level of protection might not be correlated with circulating IgG, but rather peripheral located antibodies.

These findings suggest that increased protection might be achieved with mucosal administration rather than traditional parenteral administration.

## 5. The Potential Role of T Cells in Designing a Vaccine for COVID-19

T cells, especially CD4^+^ and CD8^+^, are crucial in eliciting a specific and adequate immune response and producing a long-term immunological memory. A concise review by Grifoni et al. shed light on the importance of T cell response in COVID-19 and its potential in developing a new vaccine. Essentially, they looked up the antigens that the virus-specific T cells reacted to in exposed COVID-19 patients and compared them to healthy individuals. The team found that 100% of the exposed patients had CD4^+^ T cells that responded to the S protein, N protein, and M protein. This is an interesting finding as adding the M protein and the N protein to the vaccines with the S protein increases the chance of mimicking a natural COVID-19 infection, thereby eliciting a better immune response in the case of a potential future infection. As for the CD8^+^ response, the team found that, although the S protein and M protein were strongly recognized by CD8^+^ T cells, they were not a dominant target. They also found that the N protein and two other viral proteins, ORF3a and nsp6, comprised an average of 50% of the total CD8^+^ T cell response [66]. This indicates that we may be restricted in the types of antigens that can elicit a CD8^+^ T cell response. Another study by Peng et al. tested the immune memory of the T cell response of 42 recovered patients compared with healthy controls. The team found that the T cell response was significantly higher in severe compared with mild COVID-19 patients, with a significant response to the viral proteins S protein, M protein, and ORF3a. Moreover, they identified 39 separate peptides containing CD4^+^ and CD8^+^ epitopes [67]. These findings showcase the importance of considering other viral proteins, such as the N protein and the M protein, to design a vaccine for COVID-19.

## 6. Human Trials on MERS and SARS

Despite the numerous pre-clinical studies testing various vaccine candidates in animal models, only a few have reached clinical trials in humans (summarized in Table 1).

Two SARS-CoV vaccine phase I trials have been completed, evaluating the safety and immunogenicity of an inactivated SARS-CoV vaccine and a DNA vaccine expressing the spike protein [68,69]. Both vaccines were safe and well-tolerated in the study groups and showed some degree of immunogenicity.

The inactivated vaccine induced specific antibodies in 100% of participants after two doses; however, the genometric mean titer of nAb decreased after only four weeks.

The second study, conducted in the United States in 2004, tested the safety and immunogenicity of a recombinant DNA plasmid vaccine (VRC-SRSDNA015-00-VP), which expresses the S protein of SARS-CoV (enrollment: 10 participants). The results were published in 2008, showing that the vaccine generated a CD4^+^ T cell response in all recipients, along with a neutralizing antibodies response in 80% of the recipients. Unfortunately, the vaccine was only able to provide a CD8^+^ T cell response in 20% of the recipients [68].

The first human trial evaluating vaccine safety and immunogenicity of a MERS vaccine was conducted in the United States. It was a Phase I, open-label, dose-ranging trial (NCT02670187) that tested a DNA plasmid vaccine expressing the S protein of MERS-CoV (GLS-5300) [70]. The published results indicated that the GLS-5300 was well tolerated. Seroconversion, as measured by S1-ELISA, occurred in 86% of participants after two vaccinations, which then increased to 94% after three vaccinations. T cell responses were detected in 71% and 76% of participants, evaluated by ELISpot after two and three vaccinations, respectively, and neutralizing antibodies were seen in 50% of the participants. The response persisted when re-tested one year later [70].

A recently published phase I trial (NCT03399578) in the United Kingdom concluded a chimpanzee adeno vector expressing MERS Spike (ChAdOx1 MERS) vaccine was safe and well-tolerated in different doses [71]. They saw a significant increase in both T cells and IgG after vaccination, and 44% of participants had neutralizing antibodies in the group receiving the highest dose. A study investigating the effect of a two-dose vaccine regiment will clarify whether or not this will enhance the nAb response. Importantly, pre-existing T cell or antibody response did not affect the response. Still, the authors underline that the results must be evaluated with caution because of its small sample size and design. The same vaccine is being tested in a Phase I clinical trial in Saudi Arabia, enrolling 24 participants in an open-labeled, non-randomized clinical study (NCT04170829). In Russia, an ongoing Phase I/II open-dose, prospective randomized clinical trial is testing the safety and immunogenicity of an adenoviral-based vaccine, MERS-BVRS-GamVac, in 162 participants (NCT04130594).

The first in-human Phase I trial (NCT03615911) was conducted in Germany in 2018. The researchers tested the safety, efficacy, and immunogenicity of the vaccine candidate MVA-MERS-S, which is a modified vaccinia virus ankara (MVA) vector expressing the S protein of MERS-CoV [72]. In a homologous prime-boost immunization schedule in 26 participants, no severe adverse events were reported. Following the second immunization, 75% of low dose and 100% of high dose participants showed seroconversion, as measured by S1 ELISA. Neutralizing antibodies were detected after the second immunization; however, they decreased to pre-study levels after six months. T cell responses were detected in 83% and 91% of the subjects depending on dose and showed that MERS-CoV Spike specific secretion of IFN-γ predominantly came from CD8^+^ T cells. The response also decreased over time. Pre-existing immunity against the vector was observed; however, it did not correlate with antibody response.

**Table 1:** Different vaccine platforms and their respective trials for severe acute respiratory syndrome (SARS) and Middle East respiratory syndrome (MERS) vaccines.

				SARS		
Vaccine Platform	Antigen	Administration Method	Country	Trial Phase	Main Primary Outcome Measured	Estimated Study Completion Date or Results
DNA	Spike gene with truncated cytoplasmic domain	I.M, needle free injection management system	US (*n* = 10)	Phase I [68]	Safety and immunogenicity	Safe and well tolerated. CD4^+^ responses detected in all participants, nAb detected in 80% and CD8^+^ responses in 20%
Inactivated virus	Whole virion	I.M	China (*n* = 36)	Phase I [69]	Safety and immunogenicity	Safe and well tolerated. All developed nAb. Peak titer around 2 weeks, but decrease 4 weeks later
				**MERS**		
**Vaccine Platform**	**Antigen**	**Administration Method**	**Country**	**Trial Phase**	**Main Primary Outcome Measured**	**Estimated Study Completion Date or Results**
Modified vaccinia virus ankara (MVA) vector	Spike	I.M.	Germany (*n* = 26)	Phase I [72]	Safety and immunogenicity	Safe and well tolerated. 100% S1 Ab and 83–91% T cell response after two immunizations. Development of nAb, but decrease to pre-study levels after 6 months
DNA	Spike	I.M and E.P	US (*n* = 75)	Phase I [70]	Safety and immunogenicity	Safe and well tolerated. 94% developed S1 Ab and 76% developed T cell response after three immunizations. nAb was seen in 50%
Ad-vector	Spike	I.M.	UK/Saudi Arabia (*n* = 43/24)	Phase I, (NCT03399578/NCT04170829)	Safety and immunogenicity	UK: Safe and well tolerated. Able to generate T cell response as well as IgG. 44% in one group had nAb
Ad-vector	n.m	I.M.	Russia (*n* = 162)	Phase I/II, (NCT04130594)	Safety and immunogenicity	December 2020

n.m = not mentioned, I.M = Intramuscular, E.P = Electroporation, nAb = neutralizing antibodies.

## 7. Current COVID-19 Trials

The COVID-19 pandemic’s significant global impact has advanced vaccine development at an unprecedented speed. A tremendous scientific effort supported by various regulatory and financial agencies has made it possible to accelerate the development, and the results from the first clinical vaccine trial have been reported within only five months [73]. According to WHO, on 27 May 2020, 10 vaccine candidates are now in different clinical phases, and 123 vaccines are being evaluated in pre-clinical models [74]. Besides, other previously approved vaccines are being tested for their possible pan-protective effect thanks to their ability to induce non-specific interferon responses.

The following active trials were taken from Clinicaltrials.gov and WHO database. Only trials testing active vaccines are included (summarized in Table 2).

### 7.1. Clinical Trials Using Non-Replicating Viral Vectors

Two different vaccines utilizing viral vectors are currently in clinical trial.

They are Phase I (NCT04313127) and Phase II (NCT04341389), randomized, double-blinded, and placebo-controlled clinical trials in Wuhan, China. They are evaluating the safety and immunogenicity of an Adenovirus type 5 vector (Ad5-nCoV) encoding the full-length S protein of SARS-CoV-2 in 108 healthy adults 18 years and older were evaluated [73]. While most participants reported mild to moderate adverse events, including fever, muscle, or joint pain, most were transient and self-limiting. The vaccine was able to induce both humoral and T cell responses with 50–75% showing nAb, at day 28, and 83–97% positive responders using ELISpot, an assay that quantitively quantitatively measures the frequency of cytokine secretion for a cell. The T cell response was further evaluated, and both CD4^+^ and CD8^+^ responses were noted, with polyfunctional phenotypes increasing with a dose of vaccine. However, diminished responses were observed owing to the presence of pre-existing anti-Ad5 immunity.

A Phase I/II single-blinded, randomized multicenter study in the United Kingdom (NCT04324606), not yet recruiting, wants to test the safety, immunogenicity, and efficacy of the ChAdOx1 nCoV-19, similar to the previously evaluated ChAdOx1-MERS vaccine. They intend to measure the number of virologically confirmed symptomatic cases in healthy adult volunteers aged 18 to 55 years and record the incidence of a serious adverse event (estimated enrollment: 510 participants).

### 7.2. Clinical Trials Using DNA and mRNA Vaccine

A Phase I, open-label, dose-ranging trial (NCT04283461) in the United States is testing the safety and reactogenicity of a novel lipid nanoparticle (LNP)-encapsulated mRNA-based vaccine (mRNA-1273) expressing the full length S protein of SARS-CoV-2. One of the main primary outcomes of the trial is to test the frequency of solicited local reactogenicity adverse events in 45 healthy adults 18 years and older. The participants received two vaccinations, 28 days apart. The results of the trial are promising as the mRNA-1273 vaccine elicited an anti-SARS-CoV-2 immune response in all participants, with no trial-limiting safety concerns [75].

A Phase I, open-label trial in the United States (NCT04336410) is evaluating the safety, tolerability, and immunogenicity of INO-4800 on healthy volunteers between 18 and 50 years old. The vaccine will be injected intra-dermally, followed by electroporation. Using double-stranded DNA plasmids allows researchers to synthesize and code for the Spike protein, which will initiate an immune response. The primary objective of this study is to note the percentage of participants with adverse reactions post-vaccination (estimated enrollment: 40 participants).

A Phase I/II, randomized, placebo-controlled, observer-blind, dose-finding trial in the United States (NCT04368728/NCT04380701) is evaluating the safety, tolerability, immunogenicity, and efficacy of four SARS-CoV-2 RNA vaccine candidates (BNT162a1, BNT162b1, BNT162b2, and BNT162c2) in healthy adults between 18 and 85 years old. The trial is evaluating multiple primary outcome measures, such as the percentage of participants reporting local reactions, systemic events, and adverse events (estimated enrollment: 7600 participants).

### 7.3. Clinical Trials Using Inactivated Vaccine

Two Phase I/II randomized, double-blinded, placebo-controlled trial in Jiangsu, China (NCT04383574/NCT04352608) are evaluating the safety and immunogenicity of inactivated SARS-CoV-2 vaccine in healthy adults >60 or 18 to 59 years old. The trials use a formalin-inactivated and alum-adjuvanted vaccine candidate. The primary objective is to note the occurrence of adverse reactions post-vaccination and evaluate immunogenicity (estimated enrollment: 422 and 744 participants).

A Phase I/II, randomized, double-blind, placebo parallel-controlled phase I/II clinical trial in Shangqiu, China (ChiCTR2000032459) is evaluating the safety and immunogenicity of an inactivated SARS-CoV-2 vaccine in healthy individuals 3 years and older. The primary outcome measure is the incidence of adverse events.

### 7.4. Clinical Trials Using Protein Subunits

A Phase I randomized, placebo-controlled 1/2 Phase trial by Novavax in Australia (NCT04368988) is evaluating the safety and immunogenicity of a SARS-CoV-2 recombinant Spike nanoparticle vaccine with and without Matrix-M adjuvant in 131 healthy participants between 18 and 59 years old.

### 7.5. Clinical Trials Using Antigen-Presenting Cells

A Phase I clinical trial (NCT04299724), currently recruiting in China, is using inactivated artificial antigen-presenting cells expressing conserved structural and protease epitopes of SARS-CoV-2. The primary objective of the trial is to evaluate the safety of injecting COVID-19/artificial Antigen-Presenting Cells (APC) vaccine in healthy and COVID-19 positive volunteers, including all age groups from 6 months to 80 years old (estimated enrollment: 100 participants). The same institute is conducting another Phase I/II multicenter trial (NCT04276896) testing the safety and efficacy of a lentiviral minigene vaccine (LV-SMENP) of SARS-CoV-2 using modified dendritic cells and antigen-specific Cytotoxic T-Lymphocytes (CTLs) (estimated enrollment: 100 participants).

### 7.6. Clinical Trials Investigating the Protective Effects of Already Approved Vaccines

Currently, four trials are testing whether Bacillus Calmette–Guérin (BCG) vaccine for tuberculosis is an efficient way to reduce the incidence of COVID-19. The first trial is a Phase III, two-group, multicenter, open-label randomized controlled trial (RCT) (NCT04327206) in Australia, testing the COVID-19 incidence measured over the six months post-randomization (estimated enrollment: 4170 participants). The second trial is a Phase III, placebo-controlled, adaptive multicenter RCT (NCT04328441) in the Netherlands. The primary outcome measure is the number of days of healthcare workers’ absence (estimated enrollment: 1500 participants).

The vaccine is also being tested in South Africa in a Phase III, randomized, double-blinded trial (NCT04379336). The primary outcome measure is the incidence of healthcare workers’ hospitalizations owing to COVID-19 (estimated enrollment: 500 participants). Finally, there is a Phase IV, randomized, double-blind trial in the United States (NCT04348370) testing the incidence of COVID-19 infection (estimated enrollment: 1800 participants).

Several other countries have planned similar studies with BCG vaccines, modified BCG vaccines, oral polio vaccine (OPV), and the measles-mumps-rubella (MMR) vaccine, as listed on clinicaltrials.gov.

## 8. What Can We Expect from Current Vaccine Trials When Looking at the Past?

There are several things to consider when choosing a vaccine platform for the ideal vaccine candidate that will be produced and distributed around the globe. The few clinical trials for SARS and MERS might give us a hint on what to expect. Combining the results from the numerous animal studies with the few clinical trials suggests that the high immunogenicity seen in some animal models might not apply to humans. Despite the correlates of protection for SARS-CoV-2, it is believed that both a strong cellular and humeral response is necessary for full protection. Several studies have underlined the importance of nAbs [73,76].

Using an inactivated vaccine in mice models, protection from challenge was reported against both SARS and MERS; however, the protection was only partial in ferrets [37,48,77]. In humans, the response was short-lived, and if protection correlated to nAb levels, several boosts would be required for continuing protection. This follows in line with previous vaccine trials with inactivated viruses and might prove to be an inferior choice compared with other strategies. There are four inactivated vaccine trials in evaluation in China, and with different optimization, including the use of an adjuvant, they might show novel insight.

The DNA platforms were able to protect against both SARS and MERS in animal models; however, a superior response was noted combining DNA with protein in an NHP model [59]. In humans, both the SARS-DNA (VRC-SRSDNA015-00-VP) and the MERS-DNA (GLS-5300) vaccine produced both T cell responses and nAb. Yet, against SARS, the authors speculate it might not be sufficient for protection as only a minority produced CD8^+^ T cell responses [68,70]. Furthermore, if nAb are the most important players, a response of 50% would be suboptimal. A different prime-boost regime, including other platforms, might be beneficial if drawing from experience learned from animal models [59,70]. Currently, there is only one clinical trial evaluating the protective effect of a COVID-19-DNA vaccine (INO-4800) with similar approaches as seen before. While small improvements or adjustments might add to the immunogenicity, major differences compared with previous trials are probably unlikely. Nonetheless, the protective value of the generated response might be sufficient to slow the pandemic.

Considering viral vectors, Adenovirus-based vaccines have been a frequently used platform and showed promise in mice with protection using both ChAdOx1 and Ad5 [32,44,48]. However, in ferrets, incomplete protection was noted against SARS [37]. In humans, the ChAdOx1-MERS-S was able to generate a long-lived T cell and nAb response with just one injection. However, the nAb response was not reported in more than 44%, raising concern about the protective value for the broad population. Two Ad-based vaccines are currently being evaluated against COVID-19 (Ad5-nCoV and ChAdOx1-nCoV), and with the first results already published, nAb were seen in up to 75% of participants and polyfunctional CD4^+^ and CD8^+^ phenotypes, making it a promising candidate [73]. Even so, the diminished response reported owing to pre-existing immunity is a concern. The use of ChAdOx1-vector for a COVID-19 vaccine might overcome this issue; however, cross-reacting pre-existing cellular immunity should not be ignored. As different Ad serotypes circulate globally, the vaccine-generated response might vary significantly depending on geographical location [56].

The MVA vector also had promising pre-clinical data, with different groups showing a protective response against both SARS and MERS in mice models [33,45]. Still, concerns were raised about ADE in ferrets as increased liver damage was observed [41]. In humans, MVA-MERS vaccine generated both T cell responses and nAb in the majority, but the response decreased over the six months, suggesting that a vaccine formulation and regime could be optimized [72]. Currently, there are no active MVA-COVID-19 vaccine trials.

Several other approaches not previously studied for SARS and MERS are currently being evaluated in clinical trials. mRNA vaccines are advantageous for their capacity to initiate a potent immune response and minimize the risk of infection and insertion-induced mutagenesis, as well as the potential of large-scale production. Despite that, one of the common downsides of mRNA is its instability compared with DNA and its high production costs. Two different groups are evaluating mRNA-vaccines in clinical trials (mRNA-1273 and BNT162). The Moderna mRNA-1273 has been reported to be able to generate nAb-titers at similar levels to those observed in convalescent sera according to the company [78]. However, no combined results have been published, and whether or not this platform proves superior will be evaluated soon.

Vaccines using in vitro prepared specific APC have been studied extensively in cancer immunotherapy. While it has generally been proving safe, conflicting results remain about its benefits [79,80]. With the high cost and extensive preparation, this platform might be hard to distribute to the global population within a reasonable timeframe. Yet, the two trials from China might add crucial new knowledge by trying a different approach and including several epitopes in structural and non-structural genes of SARS-CoV-2.

Several countries are evaluating another alternative methodology by looking at the non-specific effect of live-virus vaccination, including the BCG vaccination. It is increasingly acknowledged that the innate immune system can display adaptive characteristics after certain infections or vaccinations somewhat comparable to the adaptive properties seen in the adaptive immune response [81]. The BCG vaccine has previously been linked to several non-specific benefits by mounting a more efficient cytokine response [82]. An encouraging study in 2011 found that upper respiratory infections were significantly decreased in an elderly population following BCG vaccination. As we see the same kind of infection in the upper respiratory tract in COVID-19, the use of BCG vaccine against SARS-CoV-2 might have some promising results [83]. Similar non-specific effects of OPV and MMR vaccinations have been reported and high MMR vaccination coverage has been linked to few COVID-19 deaths [84]. A major advantage of this approach is that the protective effect of the BCG vaccine is currently being evaluated in several Phase III/IV trials. If a beneficial tendency is seen, no further evaluations will be necessary before implementation. Whether or not this non-specific effect will be sufficient remains to be seen.

**Figure 1:**
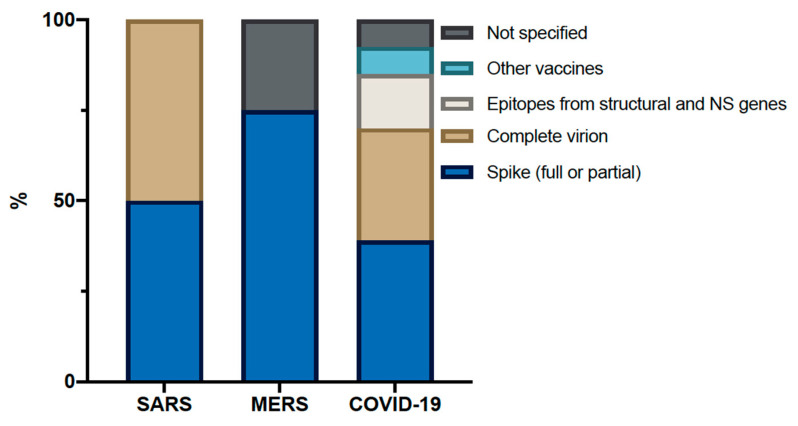
Types of antigens selected for the vaccine development (% of total registered active or completed clinical trials). SARS = severe acute respiratory syndrome, MERS = Middle East respiratory syndrome, COVID-19 = Coronavirus disease 2019, NS = Nonstructural

Interestingly, the different vaccine platforms in the clinical trials against SARS and MERS primarily use either the whole virion or *S* gene. While we can see more diversity for COVID-19, it remains dominated by the same antigens (Figure 1). One concern from animal models is the possible ADE using the full-length Spike or S1; however, this challenge was far from reported in all studies, as less pathology in vaccinated groups was frequently reported [32,41,45,77]. Despite that, if this adverse event appears, a few other non-spike candidates are in the pipeline, as listed in the pre-clinical landscape overview by WHO. Besides, another antigen selected might help guide a broader immune response. As other structural proteins appear to be more conserved between CoV, it might be an interesting approach for a pan-CoV vaccine design.

**Figure 2:**
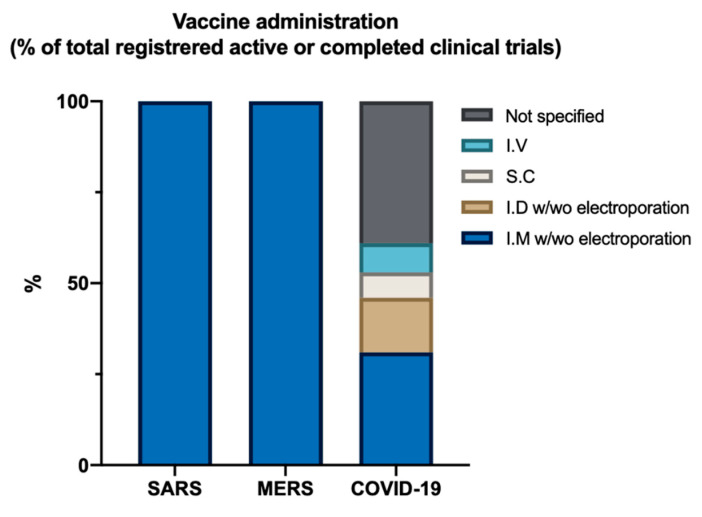
Route of vaccine administration (% of total registered active or complete clinical trials). I.M = Intramuscular, I.D = Intradermal, S.C = Subcutaneous, I.V = Intravenous.

Furthermore, no studies currently in clinical trial seem to evaluate the effect of mucosal administration, despite some pre-clinical studies showing clear evidence for improved impact (Figure 2) [44,48,50,64,65]. Nevertheless, not all trials show clear information, and some might be evaluating these administration methods. No results have been published for a Phase II trial so far, and the protective value of these vaccines in humans remains unknown. This leaves a lot of questions unanswered, even after 18 years since the SARS outbreak.

## 9. Conclusions

The last time coronavirus made its appearance was in 2012 during the MERS outbreak. Years later, and after completing only five Phase I human vaccine trials against SARS and MERS, we still have many questions unanswered. The previous outbreaks were contained primarily with non-pharmacological methods (e.g., quarantines, social distancing, masks), leaving specific prophylactic and therapeutic options as a scientific question rather than a political priority [85]. The current situation, with more than 600,000 confirmed deaths and a world in lockdown, has changed the tune, and all possible resources are now aimed at finding sustainable solutions. Since 2002, pre-clinical and clinical vaccine studies against these CoV have shown some degree of immunogenicity and protection; however, no clear-cut success story has been written. Many of the same approaches taken for SARS and MERS are being re-tested again, even though we probably can expect the same result and, for now, a lot of the suggested improvements are not being evaluated. Nevertheless, much is still under development, and information is being kept close, so hopefully some lessons have been learned.

Finding potential vaccine candidates for COVID-19 is naturally the first step taken, but subsequent vaccine production and distribution is an important subject that has previously been the bottleneck. In the 2009 H1N1 influenza outbreak, the majority of vaccine supplies were bought by wealthy nations, leaving limited stocks for low- or middle-income countries [86]. Moreover, even though the United States had the vaccine, it was distributed to other countries only after six months, which was too late, as the second wave of the virus had already begun [87]. Today, world leaders are trying to find common ground to produce and distribute the best candidates to all countries based on needs and not economic power. Still, national and financial interests might make this battle more difficult.

Most of the current clinical trials estimate to publish their results in 2021, and this means that we have at least seven months of uncertainties regarding the fate of this virus. Only time will tell whether we are on the right track to discovering the right vaccine for COVID-19.

## Figures and Tables

**Table 2:** Summary of current recruiting clinical trials for severe acute respiratory syndrome coronavirus 2 (SARS-CoV-2) found on clinicaltrials.gov and World Health Organization (WHO) database.

Vaccine Platform	Antigen	Administration Method	Country	Trial Phase	Main Primary Outcome Measures	Estimated Study Completion Date/Results
BCG	Non-SARS-CoV-2	I.D	Australia (*n* = 4170)	Phase III (NCT04327206)	COVID-19 disease incidence including symptoms and a positive SARS-CoV-2 PCR test	30 March 2022
BCG	Non-SARS-CoV-2	I.D.	Netherlands (*n* = 1500)	Phase III (NCT04328441)	Healthcare workers absenteeism	25 December 2020
BCG	Non-SARS-CoV-2	I.D.	South Africa (*n* =5500)	Phase III, (NCT04379336)	Healthcare workers morbidity and mortality	28 April 2021
BCG	Non-SARS-CoV-2	I.D	US (*n* = 1800)	Phase IV, (NCT04348370)	Healthcare workers, reduction in infection and disease severity	November 2021
Antigen presenting cells	Cons. epi	S.C	China (*n* = 100)	Phase I (NCT04299724)	Frequency of adverse events and serious adverse events and proportion of subjects with positive T cell response	31 December 2024
Lentiviral vector system	Cons. epi	S.C and I.V	China (*n* = 100)	Phase I/II (NCT04276896)	Clinical improvement based on the seven-point scale Lower Murray lung injury score	31 December 2024
Adenovirus Vector System	FL-S	I.M	China (*n* = 108)	Phase I (NCT04313127) Phase II (NCT04341389)	Adverse events and immunogenicity	Mild to moderate transient adverse events in 81% of participant. B and T cell response in all participant. Pre-existing Ad immunity diminished vaccine response
Adenovirus Vector System	FL-S	I.M for comparator, n.m for vaccine	UK (*n* = 510)	Phase I/II (NCT04324606)	Number of virologically confirmed symptomatic cases and safety	May 2021
mRNA	FL-S	I.M	US (*n* = 105)	Phase I (NCT04283461)	Safety and reactogenicity	20 September 2021
mRNA	n.m	I.M	US (*n* = 7600)	Phase I/II (NCT04368728)	Local reactions and systemic events	27 January 2023
DNA	S	I.D. and E.P	US (*n* = 40)	Phase I (NCT04336410)	Adverse events and immunogenicity	April 2021
Inactivated vaccine	Whole virion	n.m	China (*n* = 744/422)	Phase I/II (NCT04352608/NCT04383574)	Adverse events and immunogenicity	13 December 2020
Inactivated vaccine	Whole virion	n.m	China	Phase I/II (ChiCTR2000032459)	Adverse events and immunogenicity	__
Inactivated vaccine	Whole virion	n.m	China	Phase I/II (ChiCTR2000031809)	Adverse events and immunogenicity	__
Inactivated vaccine	Whole virion	n.m	China (*n* = 942)	Phase I/II NCT04412538	Adverse events and immunogenicity	__
Protein subunit	rS nano	I.M	Australia (*n* = 131)	Phase I (NCT04368988)	Adverse events and immunogenicity	31 July 2021

I.M = Intramuscular, I.D = Intradermal, S.C = Subcutaneous, I.V = Intravenous, E.P =Electroporation, n.m = not mentioned, FL = Full-Length, Cons. Epi = conserved epitopes in structural and protease genes, S = spike, nano = nanoparticle, BCG = Bacillus Calmette–Guérin, COVID-19 = Coronavirus disease 2019.
